# Altered Venous Function during Long-Duration Spaceflights

**DOI:** 10.3389/fphys.2017.00694

**Published:** 2017-09-12

**Authors:** Jacques-Olivier Fortrat, Ana de Holanda, Kathryn Zuj, Guillemette Gauquelin-Koch, Claude Gharib

**Affiliations:** ^1^UMR Centre National de la Recherche Scientifique, Faculté de Médecine d'Angers, 6214 Institut National de la Santé et de la Recherche Médicale, 1083 (Biologie Neurovasculaire et Mitochondriale Intégrée) Angers, France; ^2^Faculty of Applied Health Sciences, University of Waterloo Waterloo, ON, Canada; ^3^Centre Nationale d'Etudes Spatiales Paris, France; ^4^Faculté de Médecine Lyon Est, Université Claude Bernard Lyon 1 Lyon, France

**Keywords:** blood volume, cardiovascular deconditioning, microgravity, venous plethysmography

## Abstract

**Aims:** Venous adaptation to microgravity, associated with cardiovascular deconditioning, may contribute to orthostatic intolerance following spaceflight. The aim of this study was to analyze the main parameters of venous hemodynamics with long-duration spaceflight.

**Methods:** Venous plethysmography was performed on 24 cosmonauts before, during, and after spaceflights aboard the International Space Station. Venous plethysmography assessed venous filling and emptying functions as well as microvascular filtration, in response to different levels of venous occlusion pressure. Calf volume was assessed using calf circumference measurements.

**Results:** Calf volume decreased during spaceflight from 2.3 ± 0.3 to 1.7 ± 0.2 L (*p* < 0.001), and recovered after it (2.3 ± 0.3 L). Venous compliance, determined as the relationship between occlusion pressure and the change in venous volume, increased during spaceflight from 0.090 ± 0.005 to 0.120 ± 0.007 (*p* < 0.01) and recovered 8 days after landing (0.071 ± 0.005, arbitrary units). The index of venous emptying rate decreased during spaceflight from −0.004 ± 0.022 to −0.212 ± 0.033 (*p* < 0.001, arbitrary units). The index of vascular microfiltration increased during spaceflight from 6.1 ± 1.8 to 10.6 ± 7.9 (*p* < 0.05, arbitrary units).

**Conclusion:** This study demonstrated that overall venous function is changed during spaceflight. In future, venous function should be considered when developing countermeasures to prevent cardiovascular deconditioning and orthostatic intolerance with long-duration spaceflight.

## Introduction

In a standing posture, gravity induces peripheral venous pooling that, when excessive, can lead to orthostatic intolerance and fainting (Rowell, 1993; Fedorowski et al., 2012; Raj, 2014). For cosmonauts returning to Earth, the risk of orthostatic intolerance and fainting is greater in part due to hypovolemia resulting from adaptation to the microgravity environment (Gharib et al., 1988; Blomqvist et al., 1994; Fortney et al., 1996; Coupé et al., 2011). However, this hypovolemia is moderate and cannot fully explain the observed orthostatic intolerance in cosmonauts following spaceflight (Blomqvist and Stone, 1983; Fortney et al., 1996).

Other adaptations to microgravity have been identified that could contribute to orthostatic intolerance including changes in cardiac and baroreflex autonomic control (Shen et al., 1988; Eckberg and Fritsch, 1992; Hughson et al., 1994). While these adaptations are thought to primarily involve the arterial system, it should be noted that veins also possess autonomic adrenergic receptors that modulate functions including pooling capacity (Gelman, 2008). Previous work, using ultrasound imaging, has shown that venous morphology is altered during spaceflight (Arbeille et al., 2001, 2015). Although commonly used in daily medical practice, ultrasound imaging does not provide a global assessment venous functions (Donnelly et al., 2000). Therefore, it is still unknown whether spaceflight results in alterations to venous autonomic control or general venous function that could contribute to orthostatic intolerance.

Venous functions are complex and include factors such as filling and emptying properties, efficiency of the muscular venous pump, as well as microvascular filtration (Stewart, 2003; Krishnan et al., 2009). Venous occlusion plethysmography is one method that has been proposed to assess venous function (Skoog et al., 2015). Studies using this method have shown that the filling function of veins is altered during simulated and real short-term spaceflight (Bungo, 1989; Louisy et al., 2001; Besnard et al., 2002). Therefore, the purpose of the current study was to use this method to assess venous function with long-duration spaceflight. It was hypothesized that long duration spaceflight would result in alterations in venous function that may lead to greater pooling in an upright posture and reduced orthostatic tolerance.

## Materials and methods

### Subjects

Twenty-four male cosmonauts from the Russian space program were studied between 2009 and 2015 during spaceflights (124–192 days) aboard the International Space Station. Their mean ± *SD* anthropometric characteristics were as follows: age 44.3 ± 6.1 years, weight 82.6 ± 6.7 kg, height 1.77 ± 0.05 m, body mass index 26.4 ± 2.3 kg/m^2^. Their physical activity was not controlled before flight but each performed 2 h of exercise daily aboard the International Space Station as detailed elsewhere (Petersen et al., 2016). Data collection was performed by the medical team of the Institute for Bio-Medical Problems (IMBP, Moscow, Russia, see Section Acknowledgments) as part of the cosmonauts' regular medical supervision. This study was carried out in accordance with the recommendations of the Institutional Review Board of IMBP and all subjects gave written informed consent in accordance with the Declaration of Helsinki.

### Protocol

Data were collected on six testing days with two sessions occurring before spaceflight, two during the flight, and two after returning to Earth. The initial pre-flight session occurred more than 2 months before spaceflight (*B* > 2) with the second session occurring <2 months before the flight (*B* < 2). An early spaceflight session was conducted during the first 3 months of flight (*F* < 3) and a late flight session was conducted after the cosmonaut had spent 3 months in space (*F* > 3). Post-flight sessions occurred within 15 h of landing and 8 days after landing (L0 and L8, respectively). Each cosmonaut took self-measurements during the flight after having undergone training for the procedure before the flight. On Earth, all measurements were conducted with the cosmonaut in a supine position with the measurement leg supported at heart level according to the standard procedures (Stewart, 2003; Krishnan et al., 2009). During spaceflight, cosmonauts were in a “free floating” position with the knee slightly bent and the thigh in weak abduction.

### Calf volume

Calf volume (CV) was determined by means of calf circumference measurements (measuring tape) using the method described by Thornton et al. (1992). Briefly, nine calf circumference measurements, distributed along the leg in predetermined positions were performed. The calf section between two adjacent circumference measurements was considered to be a truncated cone in order to convert the measured distances into a volume. The calf volume was the sum of the volume of the 8 truncated cones. A calf volume measurement was repeated before every plethysmography session.

### Air plethysmography

Venous function was determined using an Air Plethysmograph APG® 1000 (ACI Corporation, San Marcos, CA, USA) that was modified for use in a microgravity environment. The device consists of a long tubular air cuff, positioned around the lower leg, that was inflated to a pressure of about 6 mmHg by an air pump. Throughout testing, the pressure in the cuff was constantly measured reflecting variations in leg volume.

**Figure 1:**
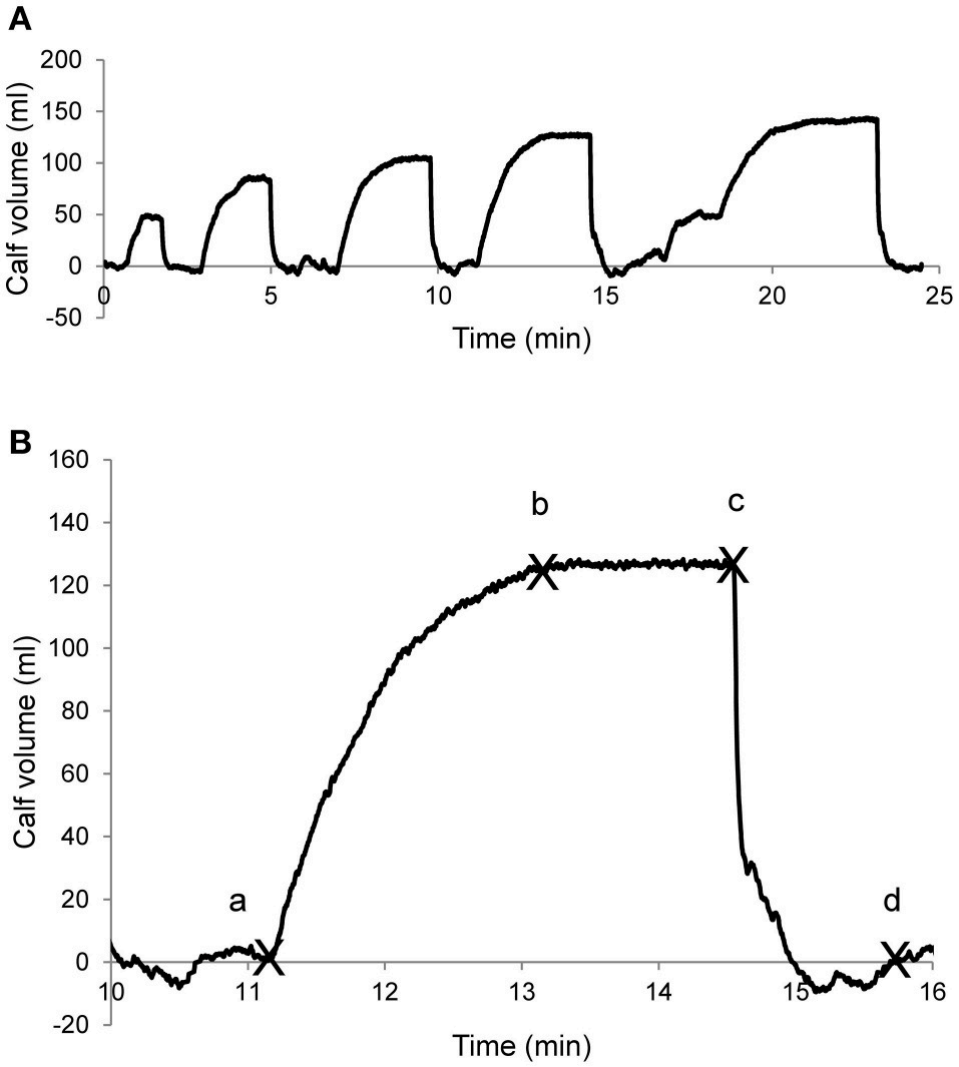
Example of the venous plethysmography curve. **(A)** A whole session that includes several venous occlusion steps at increasing pressure (20, 30, 40, 50, and 60 mmHg, from left to right). **(B)** Example of an occlusion step showing the points to determined plethysmography variables: a, b: start and end points for calf vein filling measurements; c, d: start and end points for calf vein emptying measurements.

Venous hemodynamics were assessed according to the procedure previously described (Stewart, 2003; Krishnan et al., 2009) with the determination of standard venous plethysmography parameters (Boccalon et al., 1987; Neglén and Raju, 1995; Louisy et al., 1997, 2001; Krishnan et al., 2009; Lattimer et al., 2014; Shiraishi, 2014). Venous occlusion was performed using a thigh cuff with changes in leg volume determined at five levels of venous occlusion; 20, 30, 40, 50, and 60 mmHg (Figure 1). For each level of venous occlusion, an “n” shaped curve was obtained with an increase in calf volume followed by a plateau with occlusion and a decrease in calf volume to pre-occlusion values with thigh cuff deflation (Figure 1). Venous occlusion was applied long enough to reach the plateau of the “n” shape curve as visually estimated by the operator. Venous occlusion lasted 3–5 min. Four points where marked by visual inspection by a trained operator (JOF): (a) the beginning of the volume increase, (b) the relative stabilization of the volume after the increase, (c) the beginning of the deflation, and (d) the relative stabilization of the volume after the deflation (point noted a, b, c, and d, respectively, Figure 1).

**Figure 2:**
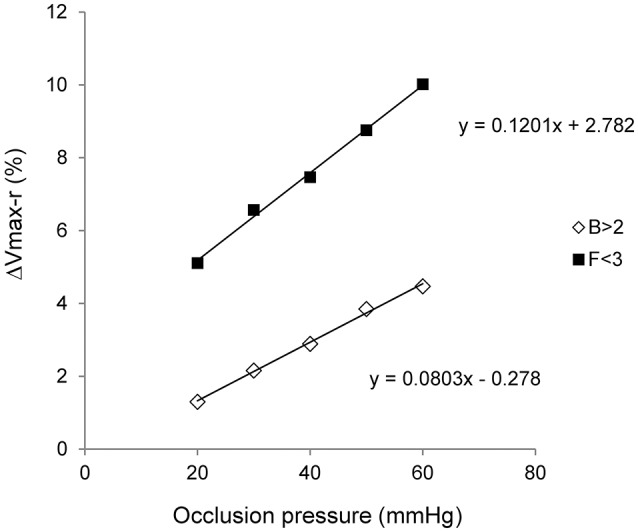
Venous compliance. Venous compliance is assessed through the pressure/volume relationship. The diagram is drawn using the relative filling volume (ΔVmax-r, in percentage) and the venous occlusion pressure during two whole plethysmography sessions on the same cosmonaut. The first session occurred more than 2 months before space flight (*B* > 2) and the second one during the flight but before its third month (*F* < 3). A whole plethysmography session included five levels of venous occlusion (x-axis). Equations of the linear regressions are mentioned on the graph.

The initial fast increase (first 20 s), linked to arterial inflow and an arterial filling velocity, was assessed as the change in calf volume over the first 20 s of venous occlusion (aV in ml/min; Louisy et al., 1997; Shiraishi, 2014). The absolute volume increase at the plateau was used as a determination of venous filling function (ΔVmax-a in ml, Louisy et al., 1997, 2001). Venous filling was also determined with respect to resting calf volume [ΔVmax-r, in percentage, that is (ΔVmax-a/CV) ^*^100] for the determination of venous capacitance (Louisy et al., 1997, 2001). This value was plotted against venous occlusion pressure (Figure 2) with the slope of the relationship providing an indication of venous compliance (Neglén and Raju, 1995; Krishnan et al., 2009,). Finally, venous distensibility was assessed as the Venous Filling Index (VFI, the mean filling velocity of 90% of the ΔVmax-a, in ml/min; Louisy et al., 2001; Shiraishi, 2014).

**Figure 3:**
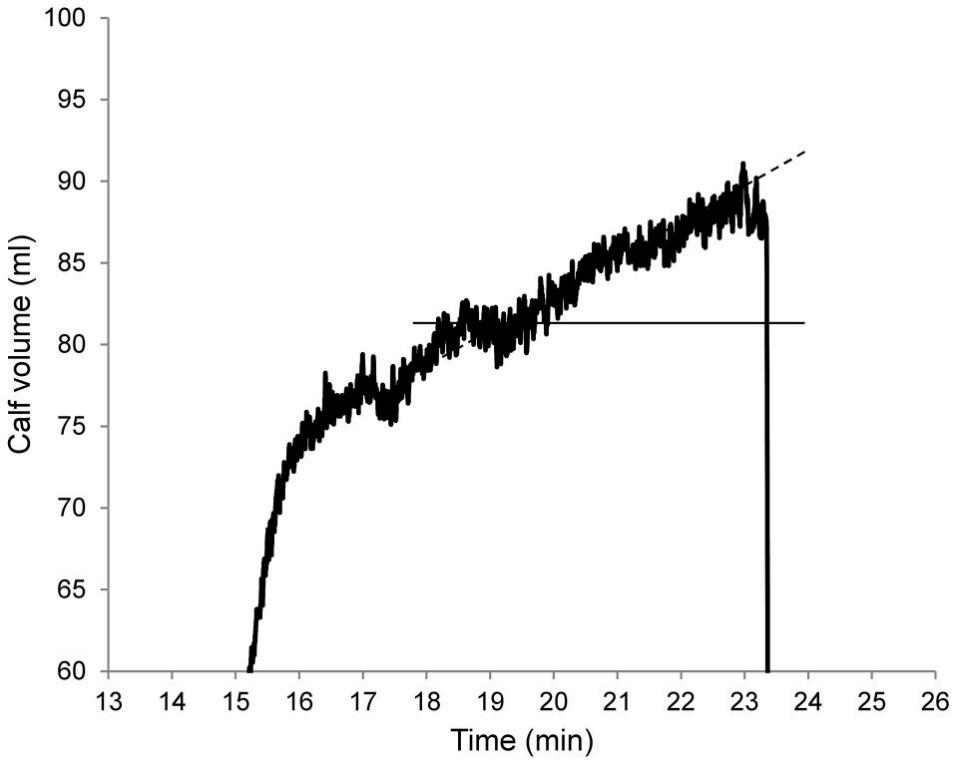
“Plateau” section of a venous plethysmography curve. This figure shows the drift of the upper plateau when the venous occlusion cuff is inflated (at 50 mmHg in this example). This drift is almost linear at the end of the plateau and this is due to microvascular filtration resulting in increased calf volume. The slope of the dashed line provides a quantification of this drift and is used to assess microvascular filtration.

The drift at the end of the plateau was quantified as the slope of the line passing through the second and the third marked points (b and c on Figure 1, arbitrary units). With the venous occlusion cuff inflated, this drift is due to microvascular filtration increasing calf volume (Stewart, 2003; Krishnan et al., 2009; Figure 3).

Venous emptying is characterized by an initial fast emptying followed by a slower emptying. The initial part, dependent on venous elasticity and resistance to venous outflow (Boccalon et al., 1987; Louisy et al., 1997), was assessed as the emptying rate of 50% of pooled venous volume (VER50%, in ml/s). The slower emptying, mainly dependent on resistance to venous outflow, was quantified as the emptying rate of 90% of pooled venous volume (VER90%, in ml/s, Lattimer et al., 2014).

### Statistics

Data are presented as means ± *SD*. Each plethysmography session was analyzed as previously described (Stewart, 2003; Krishnan et al., 2009). Briefly, values of each of the plethysmography parameters were plotted against venous occlusion pressures to determine the slopes of the regression lines (see Figure 2). Slopes of regression lines were then compared using a between-period analysis of variance (ANOVA) after a Barlett's test for equality of variances. When appropriate, a *post-hoc t*-test for paired data with Bonferroni correction was applied (Prism 5.01, GraphPad Software, San Diego, CA, USA). All cosmonauts did not perform all plethysmography sessions due to operational limitations, therefore, a repeated measurement ANOVA was not performed. Statistical significance was set at *p* < 0.05.

## Results

One hundred and three plethysmography sessions were performed during this study (Table 1). Post-flight data were only collected on a small number of cosmonauts due to the late introduction of these measures into the study and operational limitations. Calf volume measures and venous plethysmography results for each testing day are presented in Table 1.

**Table 1:** Plethysmography data during long-term spaceflight.

	***B* > 2**	***B* < 2**	***F* < 3**	***F* > 3**	**L0**	**L8**
*n*	21	20	22	24	9	9
CV (L)	2.3 ± 0.3	2.4 ± 0.3	1.7 ± 0.2^***^^+++^	1.7 ± 0.2^***^^+++^	2.1 ± 0.4^$^^§§^	2.3 ± 0.3^$$$^^§§^
aV	0.56 ± 0.0.97	0.32 ± 0.0.77	0.76 ± 1.46	0.46 ± 0.0.83	1.39 ± 1.00	0.18 ± 0.85
ΔVmax-a	2.07 ± 0.47	2.19 ± 0.53	2.25 ± 0.59	1.94 ± 0.51	1.88 ± 0.38	1.67 ± 0.56
ΔVmax-r	0.09 ± 0.02	0.09 ± 0.02	0.12 ± 0.03^**^^++^	0.11 ± 0.03^+^	0.09 ± 0.02	0.07 ± 0.01^$$$^^§§^
VFI	−0.44 ± 0.68	−0.96 ± 0.90	−0.07 ± 0.94^++^	−0.41 ± 0.42	−1.27 ± 1.01^$$^	−1.09 ± 0.52^§§^
VER50%	0.68 ± 0.51	0.76 ± 0.62	0.54 ± 0.79	0.39 ± 0.40	0.74 ± 0.38	0.88 ± 0.62
VER90%	−0.01 ± 0.11	0.04 ± 0.11	−0.14 ± 0.04^*^^++^	−0.21 ± 0.15^***^^+++^	−0.09 ± 0.13	−0.08 ± 0.08
μ filtration	6.1 ± 1.8	6.8 ± 2.6	10.6 ± 7.9^*^	9.0 ± 3.8	7.1 ± 3.2	7.4 ± 4.5

Measurements were conducted more than 2 months (B > 2) and <2 months before flight (B < 2), during the first 3 months of spaceflight (F<3), after 3 months of flight (F > 3), on landing day (L0), and 8 days after landing (L8). Table shows the values (mean ± SD) for the number of cosmonauts tested (n), calf volume (CV), arterial filling speed (aV), maximal filling volume (ΔVmax-a), maximal filling volume as a percentage of calf volume (ΔVmax-r), Venous Filling Index (VFI), emptying rate of 50% of pooled venous volume (VER50%), emptying rate of 90% of pooled venous volume (VER90%), and microvascular filtration (μ filtration). Calf volumes are reported in liters while all other values are the slopes of the regression lines between measurements and venous occlusion pressure and are reported in arbitrary units. Values that are statistically different from B > 2, B < 2, F < 3, and F > 3 are denoted by

*, +, $, and § *respectively. Single, double, and triple symbols represent statistical significance at p < 0.05, p < 0.01, and p < 0.001, respectively*.

Calf volume decreased during space flight and remained unchanged throughout the flight. Recovery to pre-flight volume began shortly after landing and was completed 8 days later. Absolute filling volume was not significantly altered during spaceflight or recovery from flight. However, relative to calf volume, venous filling significantly increased early during spaceflight and tended to remain elevated later in flight. Upon return to Earth, relative venous filling remained elevated on L0, but had recovered by L8. Venous distensibility, assessed through VFI, initially increased during the flight but recovered to pre-flight values later in flight. Following flight, VFI showed an exaggerated recovery on L0 and recovered to pre-flight values on L8.

Initial venous emptying (VER50%) was not significantly changed during or after spaceflight while the late venous emptying (VER90%) was decreased on the first flight session with a further decrease later in flight. Additionally, this parameter did not return to preflight levels on either L0 or L8. A slight increase in microvascular filtration was seen early in spaceflight but was not changed on any other testing day.

All the venous filling parameters had the same pattern of changes during space flight that was different from the one of calf volume and venous emptying parameters. This pattern showed a large change during the initial part of the space flight and a trend toward recovery of pre-flight values during the second part of the space flight (Table 1). Calf volume showed a large change that was maintained during the whole space-flight while the venous emptying parameters showed continuously increasing changes (Table 1). The pattern of change was similar between venous filling parameters and microvascular filtration (Table 1).

## Discussion

The purpose of this study was to investigate venous function before and during long-duration spaceflight. Consistent with the hypothesis, results indicated alterations in venous functions with adaptation to microgravity. Changes were seen with both venous filling and emptying but different patterns in responses were noted that did not completely parallel changes in calf volume.

Reduced calf volume leading to “bird legs” is a well-known result of spaceflight (Blomqvist et al., 1994) and was noted in the current study. It is generally believed that this reduction in calf volume is primarily due to muscular atrophy (Blomqvist et al., 1994). Vein function is strongly linked to muscle mass due to the actions of the muscle pump and the influences of muscle on venous transmural pressure (Atkov and Bednenko, 1992). However, upon return to Earth, venous function tended to recover after 8 days whereas muscle mass recovery requires additional time (6–8 weeks, Atkov and Bednenko, 1992). Moreover, evidence of lower limb muscle atrophy during spaceflight has mainly been obtained from animal studies during which animals were completely inactive and food intake was uncontrolled (Atkov and Bednenko, 1992). Today, cosmonauts perform daily exercise countermeasures and close attention is paid to food intake (Petersen et al., 2016). Recent work has alternatively focused on spinal muscle adaptation (Hides et al., 2016) as leg muscle atrophy is not readily apparent with the current countermeasures used. Therefore, it is unlikely that changes in leg muscle mass contributed to the changes in calf volume and venous function observed in this study.

Calf volume changes were likely the result of fluid shifts during spaceflight as volume was seen to rapidly recover upon return to Earth. However, venous blood shift alone cannot fully explain the large calf volume changes suggesting the involvement of tissues and interstitial volumes. In general, these fluid shifts undoubtedly influenced venous functions. However, venous function showed an adaptation to these shifts since venous function tended to recover toward pre-flights value after 3 months of spaceflight. In 1998, White and Blomqvist proposed a model to explain the initial cardiovascular adaptations to spaceflight which included a substantial redistribution of fluid and pressure throughout the body that differed from that seen in Earth based spaceflight simulations. Results from the current study and recent long-duration spaceflight investigations also support the idea of fluid redistribution throughout the body not only throughout the cardiovascular system but also within tissues and interstitial spaces (Baisch, 1994; Verheyden et al., 2010; Norsk et al., 2015).

Venous plethysmography demonstrated a decrease in VER90% that indicated a decrease in venous resistance. The decrease in venous resistance is also consistent with the overall vasorelaxation observed during space flight (Norsk et al., 2015). Alteration in autonomic nervous control of venous functions with spaceflight could explain the decrease in venous resistance. Recent studies have, however, challenged the notion of reduced sympathetic activity with spaceflight suggesting that adaptations likely reflect sympathoexcitation (Verheyden et al., 2010; Mandsager et al., 2015; Norsk et al., 2015). Norsk et al. (2015) observed that the increase in cardiac output during long duration spaceflights is more than previously observed during short duration spaceflights. Similarly, we demonstrated a decrease in venous resistance during spaceflight with a further decrease later in flight (VER90%, Table 1). Decreased venous resistance promotes venous return and might explain the increased cardiac output observed by Norsk et al. (2015). Alteration of venous resistance and cardiac output are likely to be the result of the body fluid redistribution suggested by White and Blomqvist (1998).

The small but significant effect of spaceflight on microvascular filtration contrasts with the strong effects on venous filling and emptying functions. The pattern of change is, however, similar between venous filling function and microvascular filtration and the lack of change could be due to the large standard deviations of the filtrations assessment. Stewart (2003) showed that microvascular filtration function is changed in patients with a postural orthostatic tachycardia syndrome (POTS) suggesting that increased microvascular filtration could be related to the development of orthostatic intolerance. However, not all cosmonauts experience orthostatic intolerance after spaceflight and few of those with orthostatic intolerance exhibit symptoms of POTS (Coupé et al., 2011). Further studies are needed to determine whether microvascular filtration can be used as a measure for identifying cosmonauts who will experience orthostatic intolerance and POTS after long-duration spaceflight.

The current study utilized venous plethysmography for the assessment of venous function. However, complete assessments of vein function requires measurements that are difficult to conduct on Earth and even more difficult in a microgravity environment. In addition to venous plethysmography assessments, measures of venous blood volume and central, and peripheral venous pressure are also required to fully explore venous function (Gelman, 2008). While the current study demonstrated changes in venous properties with long-duration spaceflight, questions remain regarding mechanism involved and potential functional consequences of these changes.

In conclusion, the current study demonstrated that both venous filling and emptying functions are altered during long-duration spaceflight. While partially associated with changes in calf volume, the changes in venous function may indicate a redistribution of fluid unique to microgravity adaptations. Understanding changes in venous function with microgravity exposure may help in the development if future countermeasures to protect against cardiovascular deconditioning and the development of orthostatic intolerance with long-duration spaceflight.

## Author contributions

JF: Analysis; JF, Ad, and KZ: Drafting of the work. JF, Ad, KZ, GG, and CG: Data interpretation, revising the work critically for important intellectual content, and final approval of the version to be published.

### Conflict of interest statement

The authors declare that the research was conducted in the absence of any commercial or financial relationships that could be construed as a potential conflict of interest.

## References

[B1] Arbeille P. Fomina G. Roumy J. Alferova I. Tobal N. Herault S. (2001). Adaptation of the left heart, cerebral and femoral arteries, and jugular and femoral veins during short- and long-term head-down tilt and spaceflights. Eur. J. Appl. Physiol. 86, 157–168. 10.1007/s004210100473 11822475

[B2] Arbeille P. Provost R. Zuj K. Vincent N. (2015). Measurements of jugular, portal, femoral, and calf vein cross-sectional area for the assessment of venous blood redistribution with long duration spaceflight (Vessel Imaging Experiment). Eur. J. Appl. Physiol. 115, 2099–2106. 10.1007/s00421-015-3189-6 25991027

[B3] Atkov O. Y. Bednenko V. (1992). The systems of the human organism in-flight and during the readaptation period, in Hypokinesia and Weightlessness: Clinical and Physiologic Aspects, eds Atkov O. Y. Bednenko V. (Madison, CO: International University Press), 241–329.

[B4] Baisch F. J. (1994). Applied potential tomography shows differential changes in fluid content of leg tissue layers in microgravity. Adv. Space Res. 14, 359–364. 10.1016/0273-1177(94)90423-5 11537940

[B5] Besnard S. Roumy J. Tobal N. Herault S. Porcher M. Boulay J.. (2002). Venous stagnation induced by 7 days in HDT, in the cerebral, ophthalmic, renal and splanchnic territories. J. Gravit. Physiol. 9, 75–76. 14977000

[B6] Blomqvist C. G. Buckey J. C. Gaffney F. A. Lane L. N. Levine B. D. Watenpaugh D. A. (1994). Mechanisms of post-flight orthostatic intolerance. J. Gravit. Physiol. 1, 122–124. 11538739

[B7] Blomqvist C. G. Stone H. L. (1983). Cardiovascular adjustments to gravitational stress, in Handbook of Physiology. The Cardiovascular System, eds Shepard J. T. Abboud F. M. (Bethesda, MD: Am. Physiol. Soc), 1025–1063.

[B8] Boccalon H. Ginestet M. C. Longhi R. Puel P. (1987). Variations de la pléthysmographie veineuse chez le sujet normal. Etude par pléthysmographie posturale et à occlusion veineuse. J. Mal. Vasc. 12, 150–157. 3585184

[B9] Bungo M. W. (1989). The cardiopulmonary system, in Space Physiology and Medicine, eds Nicogossian A. E. Leach Huntoon C. Pool S. L. (Baltimore, MD: Lippincott Williams and Wilkins), 176–201.

[B10] Coupé M. Yuan M. Demiot C. Bai Y. Q. Jiang S. Z. Li Y. Z.. (2011). Low-magnitude whole body vibration with resistive exercise as a countermeasure against cardiovascular deconditioning after 60 days of head-down bed rest. Am. J. Physiol. 301, R1748–R1754. 10.1152/ajpregu.00234.2011 21900640

[B11] Donnelly R. Hinwood D. London N. J. (2000). ABC of arterial and venous disease. Non-invasive methods of arterial and venous assessment. Brit. Med. J. 320, 698–701. 10.1136/bmj.320.7236.698 10710584 PMC1117713

[B12] Eckberg D. L. Fritsch J. M. (1992). Influence of ten-day head-down bed rest on human carotid baroreceptor-cardiac reflex function. Acta Physiol. Scand. 144, 69–76. 1509895

[B13] Fedorowski A. Franceschini N. Brody J. Liu C. Verwoert G. C. Boerwinkle E.. (2012). Orthostatic hypotension and novel blood pressure-associated gene variants: genetics of postural hemodynamics (GPH) consortium. Eur. Heart J. 33, 2331–2341. 10.1093/eurheartj/ehs058 22504314 PMC3442958

[B14] Fortney S. M. Schneider V. S. Greenleaf J. E. (1996). The physiology of bed rest, in Handbook of Physiology. Environmental Physiology, eds Fregly M. J. Blatteis C. M. (New York, MA: Oxford University Press), 889–942.

[B15] Gelman S. (2008). Venous function and central venous pressure: a physiologic story. Anesthesiology 108, 735–748. 10.1097/ALN.0b013e3181672607 18362606

[B16] Gharib C. Maurice M. Geelen G. Gauquelin G. Vincent M. Pottier J. M. (1988). Volume regulating hormones during weightlessness and simulated weightlessness, in Angiology, ed Boccalon H. (Paris: John Libbey Eurotext), 657–667.

[B17] Hides J. A. Lambrecht G. Stanton W. R. Damann V. (2016). Changes in multifidus and abdominal muscle size in response to microgravity: possible implications for low back pain research. Eur. Spine J. 25 (Suppl. 1), 175–182. 10.1007/s00586-015-4311-5 26582165

[B18] Hughson R. L. Yamamoto Y. Maillet A. Fortrat J. O. Pavy-Le Traon A. Butler G. C. (1994). Altered autonomic regulation of cardiac function during head up tilt after 28 day head down bedrest with countermeasures. Clin. Physiol. 14, 291–304. 8026146 10.1111/j.1475-097x.1994.tb00386.x

[B19] Krishnan U. S. Taneja I. Gewitz M. Young R. Stewart J. (2009). Peripheral vascular adaptation and orthostatic tolerance in Fontan physiology. Circulation 120, 1775–1783. 10.1161/CIRCULATIONAHA.109.854331 19841302 PMC2791509

[B20] Lattimer C. R. Kalodiki E. Kafeza M. Azzam M. Geroulakos G. (2014). Quantifying the degree graduated elastic compression stockings enhance venous emptying. Eur. J. Vasc. Endovasc. Surg. 47, 75–80. 10.1016/j.ejvs.2013.10.020 24268516

[B21] Louisy F. Cauquil D. Andre-Deshays C. Schroiff P. Lazerges M. Lafaye C.. (2001). Air plethysmography: an alternative method for assessing peripheral circulatory adaptations during spaceflights. Eur. J. Appl. Physiol. 85, 383–391. 10.1007/s004210100426 11560095

[B22] Louisy F. Schroiff P. Güell A. (1997). Changes in leg vein filling and emptying characteristics and leg volumes during long-term head-down bed rest. J. Appl. Physiol. 82, 1726–1733. 9173933 10.1152/jappl.1997.82.6.1726

[B23] Mandsager K. T. Robertson D. Diedrich A. (2015). The function of the autonomic nervous system during spaceflight. Clin. Auton. Res. 25, 141–151. 10.1007/s10286-015-0285-y 25820827 PMC4465859

[B24] Neglén P. Raju S. (1995). The pressure/volume relationship of the calf: a measurement of vein compliance? J. Cardiovasc. Surg. 36, 219–224. 7629204

[B25] Norsk P. Asmar A. Damgaard M. Christensen N. J. (2015). Fluid shifts, vasodilatation and ambulatory blood pressure reduction during long duration spaceflight. J. Physiol. 593, 573–584. 10.1113/jphysiol.2014.284869 25774397 PMC4324706

[B26] Petersen N. Jaekel P. Rosenberger A. Weber T. Scott J. Castrucci F.. (2016). Exercise in space: the European Space Agency approach to in-flight exercise countermeasures for long-duration missions on ISS. Extrem. Physiol. Med. 5:9. 10.1186/s13728-016-0050-4 27489615 PMC4971634

[B27] Raj S. R. (2014). How did the simple faint get so complicated? Syncope in 2014. Auton. Neurosci. 184, 1–2. 10.1016/j.autneu.2014.07.003 25042646 PMC4151883

[B28] Rowell L. B. (1993). Passive effect of gravity, in Human Cardiovascular Control, ed Rowell L. B. (New York, MA: Oxford University Press), 3–36.

[B29] Shen X. Y. Sun Y. Z. Xiang Q. L. Meng J. G. Xu L. H. Yan X. X. (1988). The study of baroreceptor reflex function before and after bed rest. Physiologist 31, s22–s23.

[B30] Shiraishi Y. (2014). Relationship between arterial inflow rate and venous filling index of the lower extremities assessed by air plethysmography in subjects with or without axial reflux in the great saphenous vein. Ann. Vasc. Dis. 7, 306–311. 10.3400/avd.oa.14-00028 25298834 PMC4180694

[B31] Skoog J. Zachrisson H. Lindenberger M. Ekman M. Ewerman L. Länne T. (2015). Calf venous compliance measured by venous occlusion plethysmography: methodological aspects. Eur. J. Appl. Physiol. 115, 245–256. 10.1007/s00421-014-3009-4 25272971

[B32] Stewart J. M. (2003). Microvascular filtration is increased in postural tachycardia syndrome. Circulation 107, 2816–2822. 10.1161/01.CIR.0000070951.93566.FC 12756156

[B33] Thornton W. E. Hedge V. Coleman E. Uri J. J. Moore T. P. (1992). Changes in leg volume during microgravity simulation. Aviat. Space Environ. Med. 63, 789–794. 1524535

[B34] Verheyden B. Liu J. Beckers F. Aubert A. E. (2010). Operational point of neural cardiovascular regulation in humans up to 6 months in space. J. Appl. Physiol. 108, 646–654. 10.1152/japplphysiol.00883.2009 20075261

[B35] White R. J. Blomqvist C. G. (1998). Central venous pressure and cardiac function during spaceflight. J. Appl. Physiol. 85, 738–746. 9688754 10.1152/jappl.1998.85.2.738

